# Epidural Stimulation of the Lumbosacral Spinal Cord Improves Trunk Lean Distances in Individuals with Cervical Spinal Cord Injury

**DOI:** 10.3390/biomedicines13020394

**Published:** 2025-02-06

**Authors:** Kundan Joshi, Nyah Smith, Enrico Rejc, Beatrice Ugiliweneza, Susan J. Harkema, Claudia A. Angeli

**Affiliations:** 1 Kentucky Spinal Cord Injury Research Center, University of Louisville, Louisville, KY 40202, USA; kundan.joshi@louisville.edu (K.J.); enrico.rejc@uniud.it (E.R.); beatrice.ugiliweneza@louisville.edu (B.U.); susan.harkema@louisville.edu (S.J.H.); 2 Department of Bioengineering, University of Louisville, Louisville, KY 40292, USA; 3 Kessler Foundation, West Orange, NJ 07052, USA; 4 Department of Medicine, University of Udine, 33100 Udine, Italy; 5 Department of Anatomical Sciences and Neurobiology, University of Louisville, Louisville, KY 40292, USA; 6 Department of Neurological Surgery, University of Louisville, Louisville, KY 40202, USA; 7 Frazier Rehabilitation Institute, University of Louisville Health, Louisville, KY 40202, USA

**Keywords:** spinal cord injury, epidural stimulation, sitting postural control, trunk kinematics

## Abstract

**Background/Objectives**: Preliminary observations support the view that spinal cord epidural stimulation (scES) combined with trunk-specific training can improve trunk stability during functional activities in individuals with thoracic spinal cord injury (SCI). We studied the acute effects of trunk-specific stimulation on sitting postural control. **Methods**: Twenty-three individuals with severe cervical SCI were implanted with an epidural stimulator. Postural control was assessed before any activity-based training, without and with trunk-specific scES. In particular, participants performed sitting with upright posture, forward/back lean, and lateral lean activities while sitting on a standard therapy mat. Full-body kinematics and trunk electromyography (EMG) were acquired. Anterior-posterior and lateral trunk displacement along with trunk velocity in all four directions were obtained and used to classify postural control responses. **Results**: Compared to no stimulation, application of trunk-specific scES led to trunk anterior–posterior displacement increases during forward/back lean (2.79 ± 0.97 cm; *p*-value = 0.01), and trunk lateral displacement increases during lateral lean (2.19 ± 0.79 cm; *p*-value = 0.01). After digital filtering of stimulation artifacts, EMG root mean square amplitudes for bilateral external oblique, rectus abdominus, and erector spinae muscles were higher with stimulation for all activities (all *p*-values < 0.03). **Conclusions**: The results indicate improvements in trunk lean distances and muscle activation when leaning activities are performed with trunk-specific epidural stimulation.

## 1. Introduction

Traumatic spinal cord injury (SCI) is characterized by loss of sensorimotor control below the level of injury. Depending on severity and level of injury, individuals with SCI may suffer from paralysis, bowel, and bladder dysfunction, and/or cardiovascular deficits [1]. Cervical SCI, with a 50% prevalence, affects upper extremities and trunk control [2]. Re-gaining sitting trunk stability with upper extremity function is one of the top priorities of individuals with SCI [3].

Numerous studies have investigated the ability of individuals with SCI to maintain trunk stability during various tasks. Compared to non-injured controls, individuals with SCI had larger center of pressure sway in static sitting and had impairments in movement time, movement velocity, upper arm joint motion, and trunk displacement during functional reaching tasks [4,5,6,7]. This reduced ability to maintain postural control can be attributed to the lack of voluntary control of trunk muscles [8]. Recovering this ability has been associated, to some extent, with activation of paralyzed hip and trunk musculature [9]. The application of neuromuscular electrical stimulation during functional tasks has led to improved pelvic tilt and shoulder height in static sitting, as well as improved dynamic sitting balance and absolute reach distances via stimulation of hip and trunk muscles [10,11].

Spinal cord epidural stimulation (scES) has been used to re-engage spinal circuitry in individuals with motor complete SCI [12]. Optimization of scES parameters has led to the recovery of activation patterns for weight-bearing standing and locomotion [13,14]. A study by Gill et al. (2020) in two individuals with thoracic SCI found that scES led to improvements in trunk stability and increases in forward reach distance that tended to be larger than forward reach distance increases using functional electrical stimulation (FES) in individuals with low cervical or thoracic SCI reported by Triolo et al. (2013) [10,15]. Using task-specific scES, Rowald et al. (2022) also observed decreased spinal lordosis and time to perform trunk flexion and extension tasks in individuals with thoracic SCI [16]. Improvements in posture of lower spine segment from sacrum to L1 spine level of individuals with SCI were also observed with scES during sitting with upright posture [17]. However, changes in trunk control due to trunk-specific scES have not been investigated in individuals with cervical SCI. Meanwhile, as a non-invasive alternative, transcutaneous stimulation has been used to reactivate spinal circuitry at different levels of the spinal cord [18]. Targeted stimulation of T11 and L1 in individuals with cervical or thoracic SCI led to decreased center of pressure displacement, more erect posture in static sitting and a more continuous and controlled leaning movement with increased reach limits [19]. Previous studies have also investigated the effect of therapy programs, which typically involve functional tasks that target recruitment of the trunk musculature with the aim of improving trunk stability, which have led to decreased upper body sway and increased reach distances in individuals with SCI [20,21]. Trunk-specific training when combined with transcutaneous spinal stimulation led to improvements in range of motion during trunk lean, trunk rotation, and reaching tasks, but when combined with epidural stimulation did not improve reaching distances [15,22]. Prior to studying the effects of trunk-specific training in conjunction with scES, it is important to examine the acute effects of trunk-specific scES on trunk control, which previously has only been investigated in the thoracic SCI population.

Thus, this study aims to analyze the acute effects of trunk-specific scES on trunk control on individuals with chronic cervical SCI. We hypothesize that trunk-specific scES will improve sitting with upright posture and multi-directional leaning abilities compared to no stimulation.

## 2. Materials and Methods

### 2.1. Experimental Protocol

Twenty-three individuals with chronic cervical SCI (Age: 34.9 ± 11.8 yrs.; time post-injury: 8.1 ± 4.2 yrs.; injury levels: C3–C8) (Table 1) were implanted with a scES unit. Individuals were recruited to participate in a prospective randomized trial examining the effects of epidural stimulation on cardiovascular regulation. Individuals 18 years of age or older with a non-progressive SCI, at least 2 years post injury were recruited for the study. Further inclusion criteria also required inability to move below level of injury and the presence of cardiovascular dysfunction, which could include persistent low blood pressures, symptoms of autonomic dysreflexia, orthostatic hypotension, dysregulation in response to postural changes, or highly variable blood pressure in a 24-h period. As previously reported, implantation consisted of a 16-electrode array implanted at the L1-S1 spinal cord level connected to a neurostimulator (Intellis, Medtronic, Minneapolis, MN, USA) internalized in the lower back [23]. In brief, neurophysiological monitoring was used during the surgical implantation to guide the localization of the electrode array; after recovery, spatiotemporal mapping in the supine position was performed in the laboratory to identify the patterns of lower extremity muscle activation in response to stimulation site, amplitude and frequency. All participants provided written informed consent for implantation, assessments and training as described in the study protocol approved by the University of Louisville’s Institutional Review Board. All research was performed in accordance with relevant guidelines/regulations and the Declaration of Helsinki. Informed consent was also obtained to publish the information/images in an online open access publication. The study was first registered in clinical trials.gov on 16 November 2017 prior to participant enrollment (NCT03364660).

**Figure 1:**
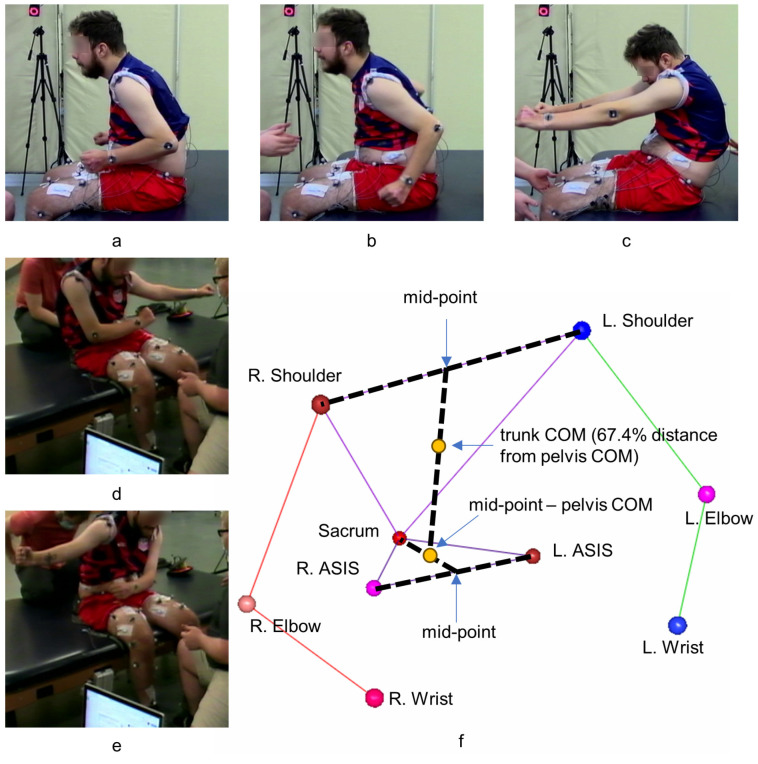
The tasks being performed: (**a**) 5-min sitting with upright posture, (**b**) forward lean phase of the forward/back trunk lean, (**c**) backward lean phase of the forward/back trunk lean, (**d**) right lean phase of the lateral trunk lean, and (**e**) left lean phase of the lateral trunk lean; (**f**) calculation of pelvis and trunk center of mass (COM) (yellow dots) using the shoulder and pelvis markers (R. Shoulder, L. Shoulder, Sacrum, R. ASIS, and L. ASIS). Note: R—Right, L—left, ASIS—anterior superior iliac spine; black dashed lines show the process of calculation of temporary virtual locations, labelled mid-points) used to estimate virtual locations of the pelvis COM and trunk COM.

All individuals were mapped and optimized for trunk-specific scES and assessed for postural control at the post-implantation stage. All individuals were mapped and optimized for trunk-specific scES and assessed for postural control at the post-implantation stage [17]. The stimulation configuration was optimized for each participant at sub-motor threshold in order to enable sitting postural control. The participants did not have any prior intervention sessions sitting with trunk-specific scES prior to the assessments. They were asked to perform three activities without upper extremity support: sitting with upright posture for 5 min, 3 attempts at forward/back trunk lean, and 3 attempts at lateral trunk lean (Figure 1a–e). All the activities were performed seated on a standard hi-lo therapy mat adjusted for height such that the participant’s hips, knees, and ankles were close to 90-degree angles. For the sitting with upright posture task, the participants were instructed to sit for 5 min whilst maintaining as upright and stable posture as possible without the support of the upper extremities. For the forward/back lean task, the participants were asked to perform a forward motion, as far as possible while maintaining control, then return to sitting with upright posture position, then a backward motion, as far as possible while maintaining control, and again return to sitting with upright posture position again. The task was repeated three times. The lateral lean task was similar to forward/back lean, with the order of the motion being left-center-right-center for 8 participants, right-center-left-center for 13 participants and both for 2 participants. At any point in these activities, if the participants tended to lose their balance, research staff provided assistance as necessary. The participants were also allowed to put their hands on the mat to ensure their safety. The assistance was removed if they regained postural control. The three postural control activities were initially performed without scES and then repeated with optimal trunk-specific scES. The participants were asked to call out at the start and end of each activity. A full-body motion capture marker set (modified Helen Hayes model) and eight Kestrel motion capture cameras (Motion Analysis Corporation, Santa Rosa, CA, USA) were used to capture kinematics. Data were acquired at 100 Hz using Cortex software Version 6.2.3.1732 (Motion Analysis Corporation, Santa Rosa, CA, USA). Bilateral Electromyography (EMG) (Motion Labs Systems Inc., Lake Elsinore, CA, USA) was acquired at 2 kHz from trunk and abdominal muscles (external oblique, rectus abdominus, erector spinae, and paraspinals) using bipolar surface electrodes. The paraspinal EMG electrodes were placed directly lateral to the implant incision site on either side in order to record the stimulation artifact, and the bilateral erector spinae EMG electrodes were placed inferior to the paraspinal electrodes.

### 2.2. Data Analysis

Marker identification in the kinematic data was performed in the Cortex software Version 6.2.3.1732 (Motion Analysis Corporation, Santa Rosa, CA, USA). Ortho Trak software Version 6.6.4 (Motion Analysis Corporation, Santa Rosa, CA, USA) was then used to generate various joint angles, and center of mass (COM) for segments of interest (Figure 1f). A custom MATLAB Version R2017b code (Mathworks, Natick, MA, USA) was used to extract COM of pelvis and trunk for the three activities. Trunk displacements in each cardinal direction (anterior, posterior, left, and right) were quantified as the maximum relative displacement of trunk COM with respect to pelvis COM in that specific direction. Then, the range between trunk anterior displacement and posterior displacement was defined as trunk anterior–posterior displacement, and the range between trunk left displacement and right displacement was defined as trunk lateral displacement. Trunk velocities in each cardinal direction were defined as the maximum of the first order derivative of the position of trunk COM in that specific direction.

For all the activities, individuals were classified as responders/non-responders to stimulation depending upon the change in resultant of peak trunk velocities, which was mathematically defined as root mean square (RMS) of trunk velocities of all four cardinal directions. Individuals were marked as responders if the decrease in resultant trunk velocity was more than a set threshold change of 1.5 cm/s or 10% of no stimulation resultant velocity, whichever was greater. Otherwise, the individuals were marked as non-responders. The peak trunk velocities were chosen to characterize the postural control responses because, during leaning tasks, a controlled movement would be associated with lower velocity, and loss of control during movement would be associated with a large increase in velocity. Similarly, during sitting with upright posture, lower velocities and displacements would indicate better postural control than higher velocities and displacements that indicate inability to maintain stable posture.

**Figure 2:**
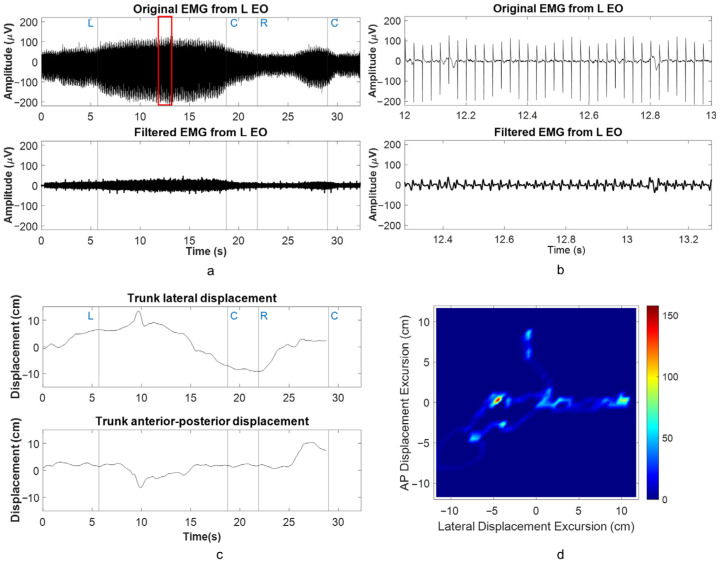
An example of filtering of L EO EMG along with trunk kinematics during an attempt at lateral trunk lean task: (**a**) overall L EO EMG during the attempt with 1-s window of data shown in red frame, (**b**) 1-s expanded window showing the effects of the filter including a time-shift of 0.275 s (550 frames), (**c**), trunk displacement during the lateral trunk lean attempt, and (**d**) trunk displacement heat map during the same attempt. Note: L-C-R-C (left-center-right-center) represents the order of callout during leaning movement of the participant.

Removal of stimulation artifact from raw EMG was carried out using empirical mode decomposition and notch filtering at the harmonics of the stimulation frequency [24]. A band-pass filter with pass band frequency of 20–150 Hz was applied prior to notch filtering to remove drifts caused by signal decomposition. An example of the effect of the filter when applied to left external oblique EMG in a lateral trunk lean attempt showed that the bursts associated with moving left/center/right/center were preserved and were analogous to trunk lateral displacement (Figure 2a–d). After filtering out the stimulation artifact, RMS amplitudes of muscle activity were then obtained for the overall sitting with upright posture or forward/back lean or lateral lean attempts for both scES OFF and scES ON conditions. However, due to the large processing time associated with the removal of the stimulation artifact from the 5-min sitting with upright posture activity, both electromyography and kinematic based outcomes were only obtained for the first one minute of the task. While application of the filter did lead to a temporal shift of ~0.275 s (550 frames) in filtered EMG, its effect on EMG RMS values over the whole attempt were considered negligible (Figure 2b). The paraspinal muscle activity was removed from the analysis as it was only intended to record the stimulation artifacts.

### 2.3. Statistical Analysis

The RMS EMG values were evaluated using mixed linear models, including a random intercept per participant to account for individual variability and random stimulation condition per participant, to account for scES ON vs. scES OFF difference variability. For forward/back trunk lean and lateral trunk lean activities where each participant had 3 attempts, a random effect for that attempt nested within stimulation condition per participant was added. Participants missing all 3 attempts for either scES ON or scES OFF or both for an outcome were excluded from the analysis of that outcome. For sitting with upright posture activity, where each participant had only one attempt, those missing any value on either scES ON or scES OFF on an outcome were excluded from the analysis of that outcome. RMS EMG were summarized using least square means and standard error from the models. The scES ON vs. scES OFF comparisons were obtained from the differences of least square means, which correspond to linear contrasts evaluated with *t*-tests. All tests were 2-sided. The significance level was set to 5%. The obtained estimate was quantified using Cohen’s d effect size, which is classified as trivial (<0.01), very small (0.01–0.19), small (0.2–0.49), medium (0.5–0.79), large (0.8–1.19), very large (1.2–1.99), and huge (≥2) by Sawilowsky’s extension of Cohen’s criteria [25,26]. Effect sizes greater than 0.5 were considered to be meaningful, as this threshold has been found to correlate with improvement in health-related quality of life [27,28]. Statistical analyses were performed in SAS 9.4 (SAS Inc., Cary, NC, USA).

## 3. Results

### 3.1. Sitting with Upright Posture

**Figure 3:**
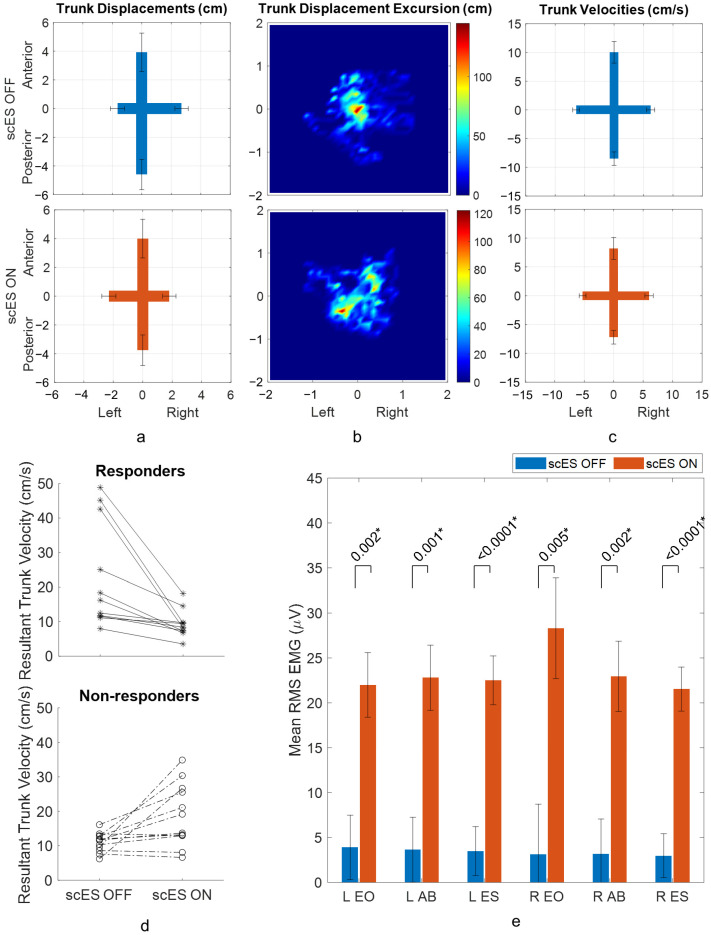
Acute responses to scES during sitting with upright posture: (**a**) trunk displacements (least square means with standard error bars) in scES OFF and scES ON conditions, (**b**) an example of trunk displacement heat map for scES OFF and scES ON conditions for participant B24, a non-responder to stimulation (color scale represents the density of the 2D displacement of the trunk with respect to pelvis), (**c**) trunk velocities (least square means with standard error bars) in scES OFF condition and scES ON conditions, (**d**) resultant maximal trunk velocity of individual participants in scES OFF and scES ON conditions for non-responders and responders to stimulation, and (**e**) RMS EMG amplitudes of trunk muscles (least square means with standard error bars) in scES OFF and scES ON conditions. * implies *p*-value is significant (<0.05).

There were no statistically significant mean differences in trunk displacements between scES OFF and scES ON conditions (Table 2, Figure 3a). An example of trunk displacement heat maps for a non-responder to stimulation showed that the participant was able to perform sitting with upright posture with trunk displacement excursion limited to ±1.5 cm in all directions in both scES OFF and scES ON conditions (Figure 3b) [29]. Similar to trunk displacements, no significant changes were observed in trunk velocities between scES OFF and scES ON conditions, even though the resultant trunk velocity maps showed that 11 out of 23 participants responded to stimulation, demonstrating lower resultant trunk velocity and greater control during sitting with upright posture (Table 2, Figure 3c,d). Five responders and 6 non-responders had resultant trunk velocity of less than 15 cm/s in both scES OFF and scES ON conditions. Among the rest of the non-responders, resultant trunk velocity was less than 15 cm/s in scES OFF condition for all but one non-responder (Figure 3d). When comparing RMS EMG amplitudes of the bilateral erector spinae, rectus abdominus, and external oblique muscles in scES OFF and scES ON conditions, statistically significant changes due to stimulation were observed in all the muscles (all *p*-values < 0.005; all Cohen’s d > 0.71) (Figure 3e).

### 3.2. Forward/Back Trunk Lean

**Figure 4:**
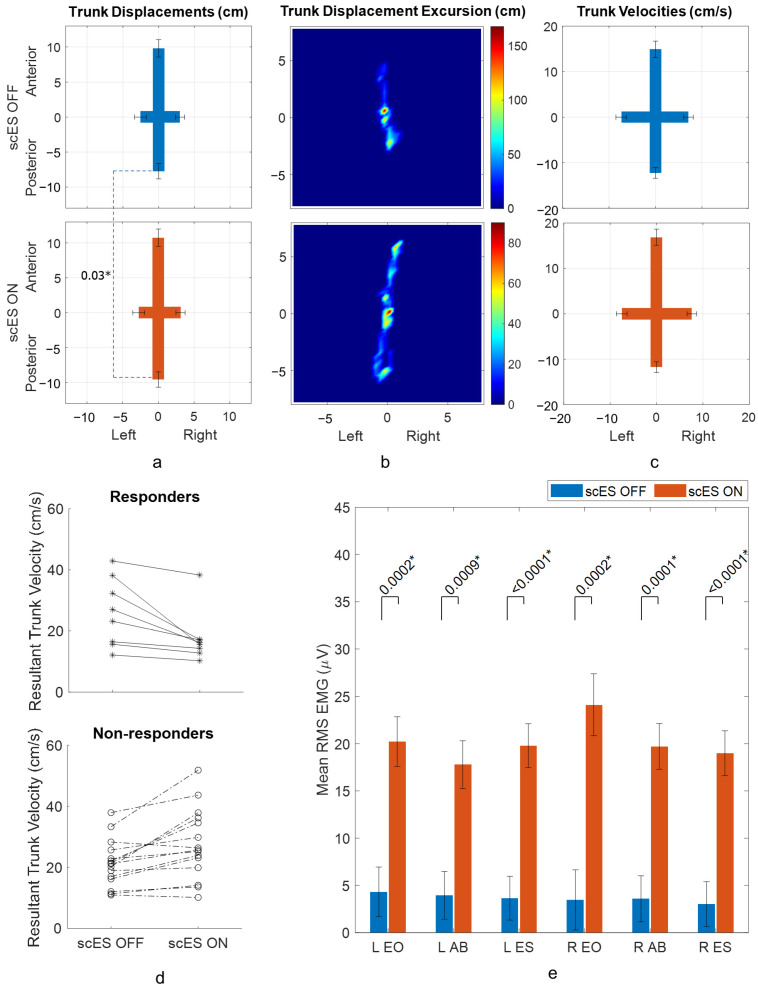
Acute responses to scES during forward/back trunk lean: (**a**) trunk displacements (least square means with standard error bars) in scES OFF and scES ON conditions, (**b**) an example of trunk displacement heat map for scES OFF condition and scES ON conditions for participant A128, a responder to stimulation, (color scale represents the density of the 2D displacement of the trunk with respect to pelvis), (**c**) trunk velocities (least square means with standard error bars) in scES OFF condition and scES ON conditions, (**d**) resultant maximal trunk velocity of individual participants in scES OFF and scES ON conditions for non-responders and responders to stimulation, and (**e**) RMS EMG amplitudes of trunk muscles (least square means with standard error bars) in scES OFF and scES ON conditions. * implies *p*-value is significant (<0.05).

When comparing trunk displacements between scES OFF and scES ON conditions in forward/back trunk lean, there was a statistically significant increase of 2.79 ± 0.97 cm (*p*-value 0.01; Cohen’s d 0.60) in trunk anterior–posterior displacement (Table 2, Figure 4a). An example of trunk displacement heat maps for a responder to stimulation showed the participant’s anterior–posterior trunk movement was unevenly spread and limited to ±4.6 cm in scES OFF condition but, in scES ON condition, the anterior–posterior trunk movement was evenly spread out to an increased range of ±6.3 cm (Figure 4b). No significant changes were observed in trunk velocity outcomes due to stimulation (Table 2, Figure 4c). The resultant trunk velocity maps showed that 8 out of 23 participants responded to stimulation and 15 participants did not (Figure 4d). For all responders except one, the resultant trunk velocity in the scES ON condition was concentrated within a range of 10.2–17.1 cm/s from a range of 12.1–38.2 s cm/s in the scES OFF condition. Among the non-responders, velocity for 4 participants did not change beyond the set responder threshold. Attempts with loss of trunk control also tended to have higher anterior and posterior velocities than controlled leaning attempts (Figure S1). When comparing RMS EMG amplitudes in scES OFF and scES ON conditions, statistically significant changes due to stimulation were observed in all the muscles (all *p*-values < 0.0009; all Cohen’s d > 0.82) (Figure 4e). Change in L ES and L AB activation was associated with change in anterior and posterior trunk velocities (Figures S2 and S3).

### 3.3. Lateral Trunk Lean

**Figure 5:**
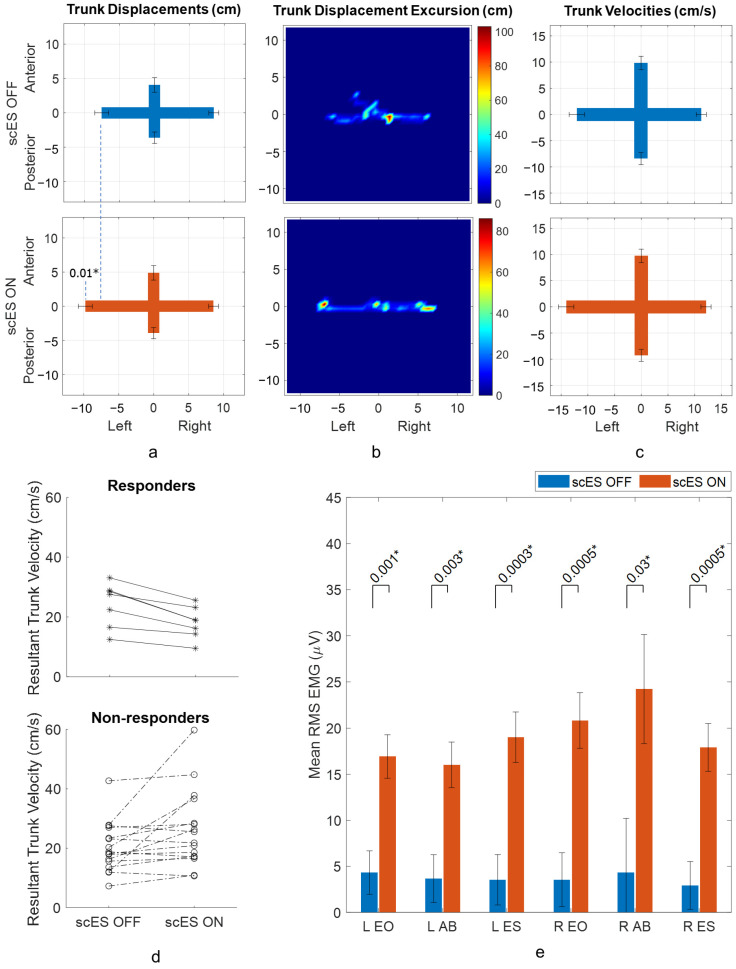
Acute responses to scES during lateral trunk lean: (**a**) trunk displacements (least square means with standard error bars) in scES OFF and scES ON conditions, (**b**) an example of trunk displacement heat map for scES OFF condition and scES ON conditions for participant C193, a responder to stimulation, (color scale represents the density of the 2D displacement of the trunk with respect to pelvis), (**c**) trunk velocities (least square means with standard error bars) in scES OFF condition and scES ON conditions, (**d**) resultant maximal trunk velocity of individual participants in scES OFF and scES ON conditions for non-responders and responders to stimulation, and (**e**) RMS EMG amplitudes of trunk muscles (least square means with standard error bars) in scES OFF and scES ON conditions. * implies *p*-value is significant (<0.05).

When comparing trunk displacements between scES OFF and scES ON conditions in lateral trunk lean, there was a statistically significant increase of 2.29 ± 1.00 cm (*p*-value 0.03; Cohen’s d 0.48) in trunk lateral displacement (Table 2, Figure 5a). An example of trunk displacement heat maps for a responder to stimulation showed the participant’s lateral trunk movement was unevenly spread up to ±6.6 cm in scES OFF condition. In scES ON condition, their lateral trunk movement was comparatively straighter and in the desired plane, with maximum displacement extending up to ±7.7 cm (Figure 5b). No significant changes were observed in trunk velocity outcomes due to stimulation (Table 2, Figure 5c). The resultant trunk velocity maps showed that 6 out of 23 participants responded to stimulation and, among the 17 non-responders, velocity for 8 non-responders did not change beyond the set responder threshold (Figure 5d). Attempts with loss of trunk control also tended to have higher left and right velocities than controlled leaning attempts (Figure S4). When comparing RMS EMG amplitudes in scES OFF and scES ON conditions, statistically significant changes due to stimulation were observed in all the muscles (all *p*-values < 0.03; all Cohen’s d > 0.50) (Figure 5e). Change in L and R ES activation was associated with change in lateral displacement. Change in L ES activation was also associated with change in left velocity. (Figures S5–S7).

## 4. Discussion

This study demonstrated that epidural stimulation of the lumbosacral spinal cord is effective at improving trunk displacement outcomes in forward/back and lateral trunk leans, while change in trunk velocities were not significant. Muscle activity measured as RMS EMG amplitudes were significantly higher with stimulation in all the activities, even though stimulation did not lead to statistically significant changes in kinematic outcomes in sitting with upright posture activity.

Ability to maintain stable sitting posture and control leaning movements are generally impaired in individuals with cervical SCI. Improvements in static sitting balance and reaching distances have been previously seen with functional electrical stimulation, transcutaneous stimulation, and epidural stimulation [11,15,19]. In static sitting with transcutaneous stimulation, Rath et al. (2018) observed decreased displacement of center of pressure than without stimulation [19]. The current study did not find overall improvement in trunk kinematics in sitting with upright posture in response to stimulation, but all abdominal and lower back muscles showed increased RMS amplitudes (all *p*-values < 0.005; all Cohen’s d > 0.71). The changes in RMS amplitude of the right erector spinae muscle (*p*-value < 0.0001; Cohen’s d 1.12) and left erector spinae muscle (*p*-value < 0.0001; Cohen’s d 1.07) were the most significant. The erector spinae muscles are responsible for spinal stability and maintenance of posture during sitting. Analogous to our results, Rath et al. (2018) also observed highest amplitude changes in erector spinae muscles at L3 level with transcutaneous stimulation in static sitting task, leading to improved spine posture [19]. Improved lower spine posture due to epidural stimulation was also observed by our group [17]. While overall trunk kinematics were not improved, individual changes were observed due to stimulation in the current study as evidenced by the resultant trunk velocity maps of the 11 responders (Figure 3d). There was a large fraction of participants, 5 responders and 11 non-responders, whose resultant trunk velocities were less than 15 cm/s without stimulation. Among them, all responders and 6 non-responders retained resultant trunk velocity less than 15 cm/s with stimulation. In addition, there were participants exhibiting a resultant trunk velocity above 25 cm/s with or without stimulation. This could be a result of spasms at either trunk or legs, leading to loss of control, marked by an increase in trunk velocities. These observed variations in individual control could have minimized the overall average change in postural control.

In multi-directional reaching, Gill et al. (2020) observed increased reach distances when epidural stimulation was applied with a significant increase in forward reach distance [15]. Bergmann et al. (2019) also observed increased flexion and lateral flexion limits when FES was delivered to erector spinae and rectus abdominus muscles [11]. Similarly, Triolo et al. (2013) observed increased reach distances with the application of FES [10]. Gauthier et al. (2013) calculated directional stability index in octagonal directions for able-bodied and SCI population using center of pressure excursions and showed statistically significant mean difference of directional stability index of 9.9 to 18.52% in six out of eight directions, while change in the index in anterior direction was not significant [30]. In the current study, in forward/back trunk lean activity, trunk anterior–posterior displacement increased from 17.54 ± 1.85 cm to 20.32 ± 1.85 cm (*p*-value 0.01; Cohen’s d 0.60) due to stimulation. The overall increase in displacement was due to trunk posterior displacement increases from 7.73 ± 1.10 cm to 9.57 ± 1.10 cm (*p*-value 0.03; Cohen’s d 0.48), while a 0.95 ± 0.66 cm increase due to stimulation in trunk anterior displacement was not significant (*p*-value 0.16; Cohen’s d 0.3). On the contrary, change due to stimulation in RMS EMG amplitudes of left erector spinae muscle (*p*-value < 0.0001; Cohen’s d 1.08), and right erector spinae muscle (*p*-value < 0.0001; Cohen’s d 1.02), which are involved in eccentric control of trunk forward lean, had larger effect sizes compared to changes in amplitudes of both the left rectus abdominus muscle (Cohen’s d 0.82) and right rectus abdominus muscle (Cohen’s d 0.98), which control trunk backward lean. While Rath et al. (2018) also observed highest amplitude changes in the erector spinae muscles at L3 level in both forward and backward lean movements with transcutaneous stimulation, the change in trunk displacement-based limits of stability was higher in the forward trunk movement compared to backward movement [19]. A possible reason behind the contrasting trend in posterior displacement was that, when stimulation was not applied, anterior displacement (9.80 ± 1.23 cm) tended to be greater than posterior displacement (7.73 ± 1.10 cm). This implied that the potential for improvement in posterior displacement was higher with stimulation. On the other hand, improvements in resultant trunk velocities were limited to individual changes with eight participants responding to stimulation, with seven out of eight responders able to obtain a velocity range between 10.2–17.1 cm/s in the scES ON condition (Figure 4d). For the participants with SCI, the decrease in velocities of their trunk, even if the difference is non-significant, would bring them closer to their goal of performing activities of daily living with higher independence [3]. Meanwhile, lateral lean distances improved with stimulation, as demonstrated by a significant increase in trunk lateral displacement from 16.04 ± 1.29 cm to 18.33 ± 1.29 cm (*p*-value 0.03; Cohen’s d 0.48). This increase was primarily due to an increase in trunk left displacement from 7.57 ± 1.00 cm to 9.76 ± 1.00 cm (*p*-value 0.01; Cohen’s d 0.58). In addition, the effect size for the change in RMS EMG amplitudes of right external oblique muscle (Cohen’s d 0.94), which is responsible for eccentrically controlling left trunk lean, was larger than the change in amplitudes of the left external oblique muscle (Cohen’s d 0.81). In contrast, Rath et al. (2018) reported greater percent increases in limits of stability in right lean movement than left lean movement along with significant increase in muscle activity in external oblique muscles only in right lean movement when transcutaneous stimulation was applied [19]. In our study, trunk left displacement (7.57 ± 1.00 cm) in the scES OFF condition tended to be higher than trunk right displacement (8.47 ± 0.71 cm), which could have been a possible reason behind the contrasting observations in trunk left displacement. On the other hand, changes in trunk velocities were not statistically significant. Individually, six participants responded to stimulation while their resultant trunk velocities in scES ON condition were not as concentrated as seen in trunk forward/back lean (Figure 4d and Figure 5d). The contrasting effects in individual responses during trunk forward/back and lateral lean could be explained by which muscle groups become activated in response to trunk-specific scES. The stimulation configurations are typically rostral on the electrode array and in a seated position the stimulation activates large muscle groups of the lower abdomen (rectus abdominus and paraspinal muscles) that are primarily involved in trunk flexion and extension. Gill et al. (2020) previously observed higher forward reach distances with caudal scES configurations that targeted the distal leg muscles than with rostral configurations (analogous to trunk-specific scES configurations in our study) [15]. However, changes in lateral reach distances were not observed with either rostral or caudal scES configurations used in their study. Lateral movement of the trunk involves activation of internal/external obliques, quadratus lumborum, and erector spinae muscles. Although increased activation of erector spinae, rectus abdominis and external oblique muscles were observed in lateral lean, obtaining improved overall control would require fine motor control and selectivity, which highlights the need for trunk-based training coupled with stimulation.

### Limitations

There has been limited past research involving trunk-specific scES and analysis of trunk kinematics outcomes. Hence, most of the comparisons were based on trunk control outcomes due to other interventions, such as transcutaneous spinal cord stimulation and/or trunk-specific therapy programs. In addition, the participant demographics in the current study were diverse in terms of age, gender, level and severity of injury, and trunk control prior to implantation. Hence, in certain outcomes, individual improvements were observed but a group change was not observed.

## 5. Conclusions

The analysis of postural control using trunk kinematics and electromyography indicated that trunk-specific spinal cord epidural stimulation led to improved lean distances in individuals with SCI, while muscle activation patterns showed greater improvements in posterior lean and left lean control. These results support our research hypothesis in multi-directional leaning tasks, even though there was not enough evidence to support it for sitting with upright posture task. Further research on rehabilitation programs that are customized to improve trunk control would be encouraged.

## Figures and Tables

**Table 1:** Participant Demographic characteristics.

ID	Gender	Age (yrs.)	Time Since Injury (yrs.)	Neuro Level	AIS
A101	M	31	2.4	C3	A
A99	M	20	2.8	C4	A
A96	F	27	3	C4	A
A102	F	29	4.4	C4	A
A123	M	29	7.8	C4	A
A105	M	34	10	C4	A
A128	M	39	8.7	C4	A
A127	F	41	13.1	C4	A
A100	M	52	16.6	C4	A
A110	F	22	5.8	C5	A
A119	F	26	11.5	C5	A
A133	M	35	13.1	C5	A
B38	M	22	3.3	C4	B
B213	F	25	5.4	C4	B
B47	M	43	8.2	C4	B
B42	M	59	7.5	C4	B
B32	M	61	7.4	C4	B
B194	F	38	18.2	C5	B
B24	M	26	6.7	C6	B
B52	F	51	8	C6	B
B41	M	27	8.6	C8	B
C193	F	41	8.9	C4	C
C231	F	27	5.7	C6	C

Note: AIS—American Spinal Injury Association Impairment Scale

**Table 2:** Differences in trunk displacements and velocities for scES OFF and scES ON conditions with Cohen’s d effect size.

	scES OFF	scES ON	Difference		Effect Size	
Task/Outcome	Mean ± SE	Mean ± SE	Mean ± SE	*p*-Value	Cohen’s d	Classification
Sitting with upright posture						
AP Disp. (cm)	8.51 ± 1.51	7.76 ± 1.51	−0.75 ± 2.13	0.73	0.07	Very Small
Lat Disp. (cm)	4.35 ± 0.45	4.11 ± 0.45	−0.24 ± 0.64	0.71	0.08	Very Small
Ant. Vel. (cm/s)	10.03 ± 1.89	8.21 ± 1.89	−1.82 ± 2.68	0.50	0.14	Very Small
Post. Vel. (cm/s)	8.49 ± 1.17	7.22 ± 1.17	−1.26 ± 1.58	0.43	0.17	Very Small
Left Vel. (cm/s)	6.45 ± 0.58	5.28 ± 0.58	−1.17 ± 0.82	0.17	0.30	Small
Right Vel. (cm/s)	6.21 ± 0.70	6.00 ± 0.70	−0.21 ± 0.98	0.83	0.05	Very Small
Forward/Back Lean						
AP Disp. (cm)	17.54 ± 1.85	20.32 ± 1.85	2.79 ± 0.97	0.01 *	0.60	Medium
Lat Disp. (cm)	5.54 ± 1.06	5.86 ± 1.06	0.32 ± 0.54	0.56	0.13	Very Small
Ant. Vel. (cm/s)	14.92 ± 1.78	16.88 ± 1.78	1.96 ± 1.78	0.28	0.23	Small
Post. Vel. (cm/s)	12.24 ± 1.17	11.76 ± 1.17	−0.48 ± 1.12	0.68	0.09	Very Small
Left Vel. (cm/s)	7.35 ± 1.15	7.48 ± 1.15	0.13 ± 1.03	0.90	0.03	Very Small
Right Vel. (cm/s)	7.11 ± 1.02	7.62 ± 1.02	0.51 ± 0.87	0.56	0.12	Very Small
Lateral Lean						
AP Disp. (cm)	7.68 ± 1.11	8.79 ± 1.11	1.12 ± 0.95	0.26	0.24	Small
Lat Disp. (cm)	16.04 ± 1.29	18.33 ± 1.29	2.29 ± 1.00	0.03 *	0.48	Small
Ant. Vel. (cm/s)	9.84 ± 1.29	9.74 ± 1.28	−0.11 ± 1.35	0.94	0.02	Very Small
Post. Vel. (cm/s)	8.42 ± 1.15	9.26 ± 1.14	0.84 ± 1.24	0.51	0.14	Very Small
Left Vel. (cm/s)	11.99 ± 1.44	14.05 ± 1.44	2.06 ± 1.45	0.17	0.30	Small
Right Vel. (cm/s)	11.25 ± 0.95	12.12 ± 0.95	0.87 ± 0.89	0.34	0.20	Small

Note: * implies *p*-value is significant (<0.05); Disp.—Displacement; Vel.—Velocity; Ant.—Anterior; Post.—Posterior; AP—Anterior–posterior; Lat—Lateral.

## Data Availability

The data presented in this study are available on request from the corresponding author. The data are not publicly available due to lack of available repository.

## Supplementary Materials

The following supporting information can be downloaded at: https://www.mdpi.com/article/10.3390/biomedicines13020394/s1. Figure S1: Peak trunk velocities in the primary anterior–posterior direction of movement for controlled attempt versus attempts with loss of control in forward/back trunk lean. Trunk anterior and posterior velocities were higher for attempts when control was lost compared to the attempts in which participants did not lose trunk control. Figure S2: Participant-wise color maps for RMS EMG amplitude changes and trunk kinematics changes between scES OFF and scES ON condition for forward/back trunk lean task. Figure S3: Scatterplot of changes during forward/back trunk lean task: (a) trunk posterior velocity versus RMS EMG amplitude of L ES, (b) trunk anterior velocity versus RMS EMG amplitude of L ES, and (c) trunk anterior velocity versus RMS EMG amplitude of L AB. 11 out of 23 participants had decrease in anterior velocity associated with increase in L AB activity (range 1.11–21.38 µA) but it was accompanied with an increase in L ES activity as well (range 1.13–62.20 µA). Eleven out of 23 participants also had decrease in posterior velocity associated with increase in L ES activity (range 1.14–20.67 µA). Figure S4: Peak trunk velocities in the primary lateral (left-right) direction of movement for controlled attempt versus attempts with loss of control in lateral trunk lean. Trunk left and right velocities were higher for attempts when control was lost compared to the attempts in which participants did not lose trunk control. Figure S5: Participant-wise color maps for RMS EMG amplitude changes and trunk kinematics changes between scES OFF and scES ON condition for lateral trunk lean task. Figure S6: Scatterplot of changes during lateral trunk lean task: (a) trunk lateral displacement versus RMS EMG amplitude of R ES, and (b) trunk left displacement versus RMS EMG amplitude of L ES. Increase in lateral displacement associated with increase in R ES activity (range 2.16–28.7 µA) for 13 out of 23 participants and with increase in L ES activity (range 2.61–43.7 µA) for 14 out of 23 participants. Figure S7: Scatterplot of changes during lateral trunk lean task: (a) trunk anterior velocity versus RMS EMG amplitude of L ES, (b) trunk anterior velocity versus RMS EMG amplitude of R ES, and (c) trunk left velocity versus RMS EMG amplitude of L ES. Decrease in anterior velocity was associated with increase in L ES activity (range 1.21–17.99 µA) and R ES activity (range 0.28–24.85 µA) for 13 out of 23 participants. Decrease in left velocity was also associated with increase in L ES activity (range 1.21–18.97 µA). Figure S8: Participant-wise color maps for RMS EMG amplitude changes and trunk kinematics changes between scES OFF and scES ON condition for sitting with upright posture task.

## Abbreviations

The following abbreviations are used in this manuscript:

scES	Spinal cord epidural stimulation
SCI	Spinal cord injury
EMG	Electromyography
FES	Functional Electrical Stimulation
COM	Center of Mass
RMS	Root Mean Square
