# Supplementary figures and images for: Epidural Stimulation of the Lumbosacral Spinal Cord Improves Trunk Lean Distances in Individuals with Cervical Spinal Cord Injury
Source: Biomedicines. 2025 Feb 6;13(2):394. doi: 10.3390/biomedicines13020394 (PMC11853460; doi:10.3390/biomedicines13020394)

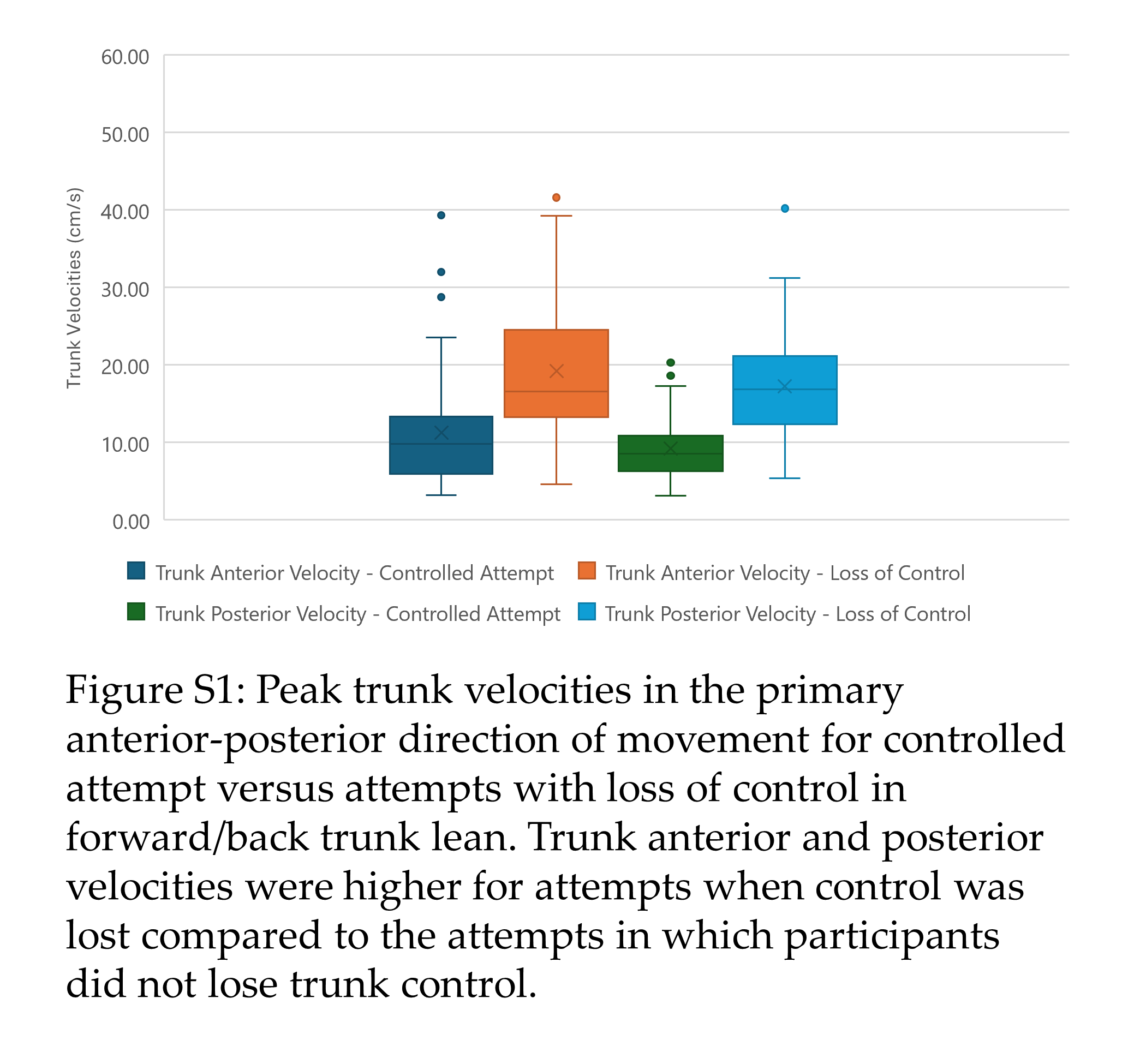

Supplement: Supplementary file 1 [file biomedicines-13-00394-s001.zip › Figure S1.TIF]

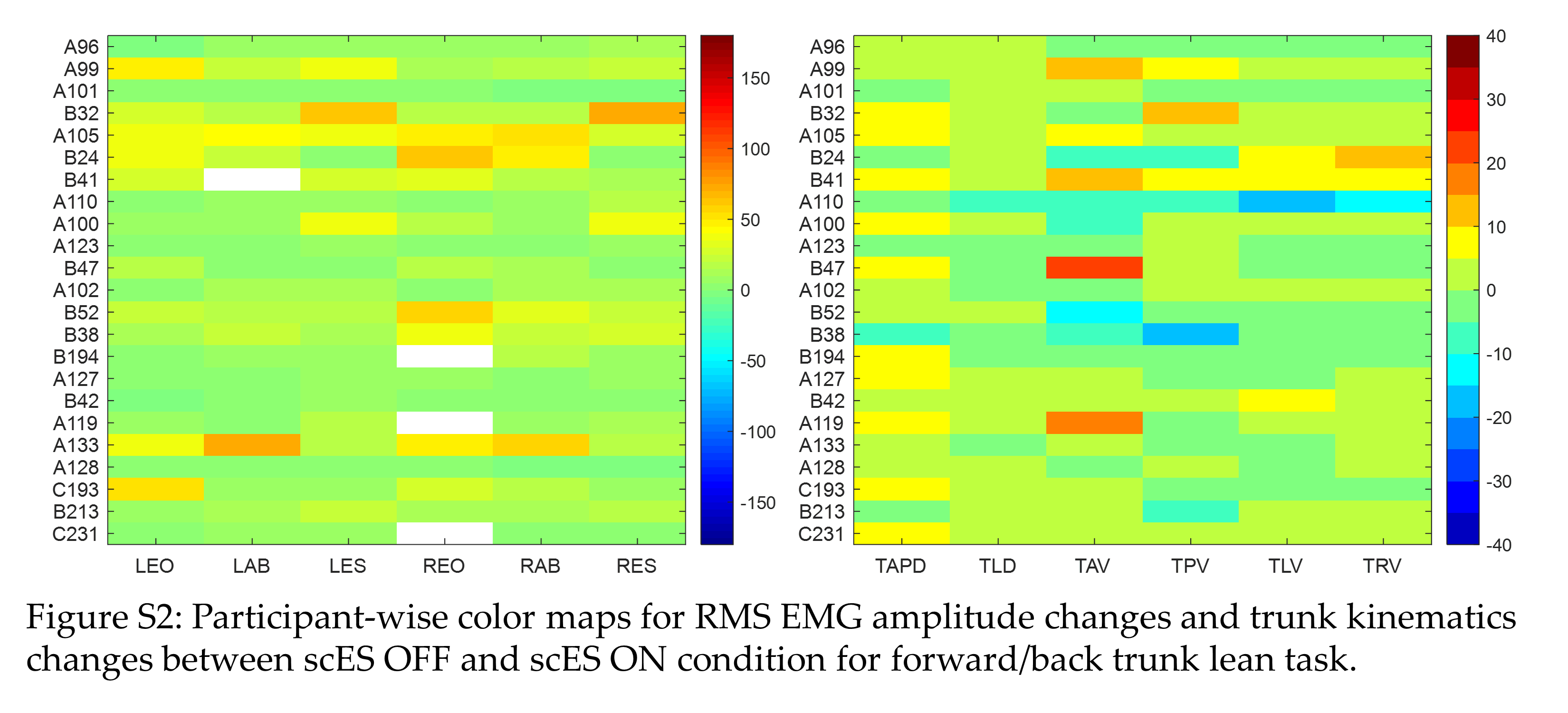

Supplement: Supplementary file 1 [file biomedicines-13-00394-s001.zip › Figure S2.TIF]

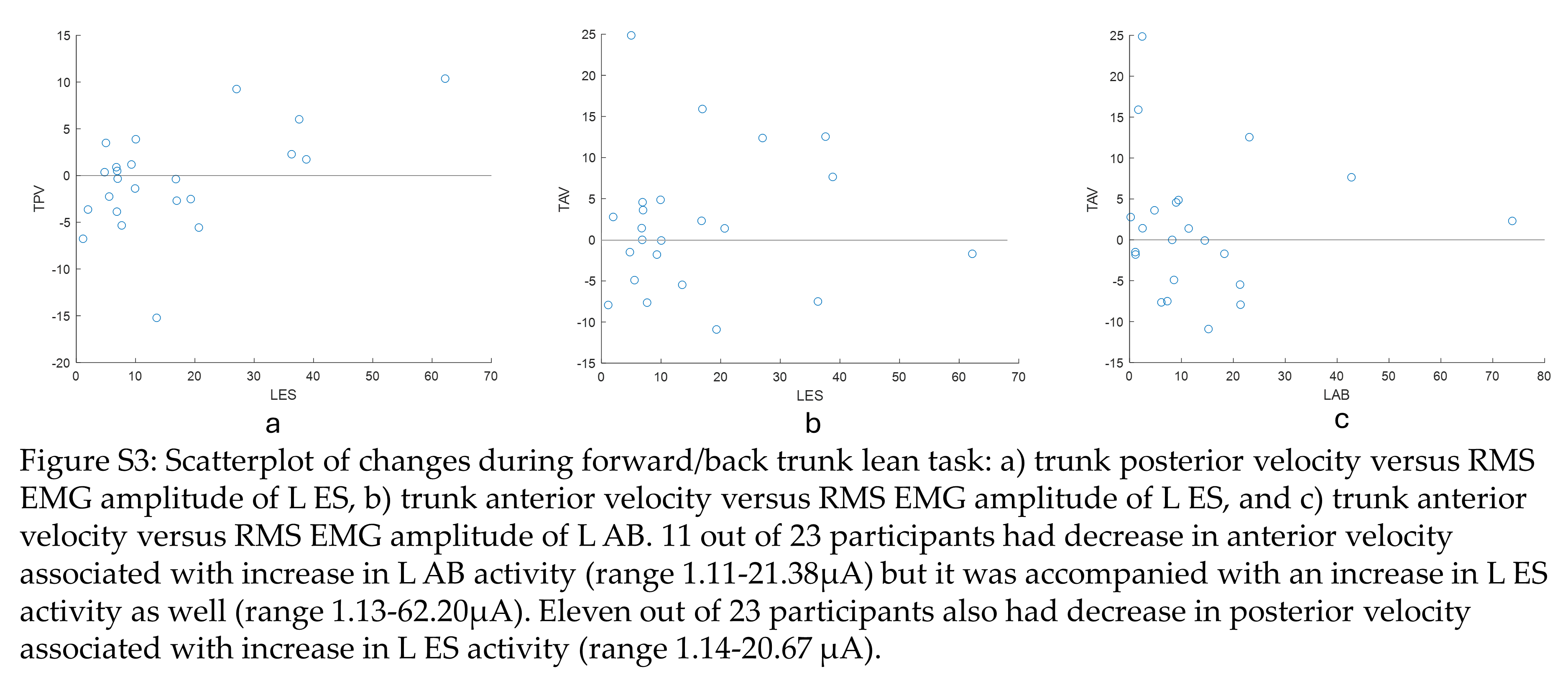

Supplement: Supplementary file 1 [file biomedicines-13-00394-s001.zip › Figure S3.TIF]

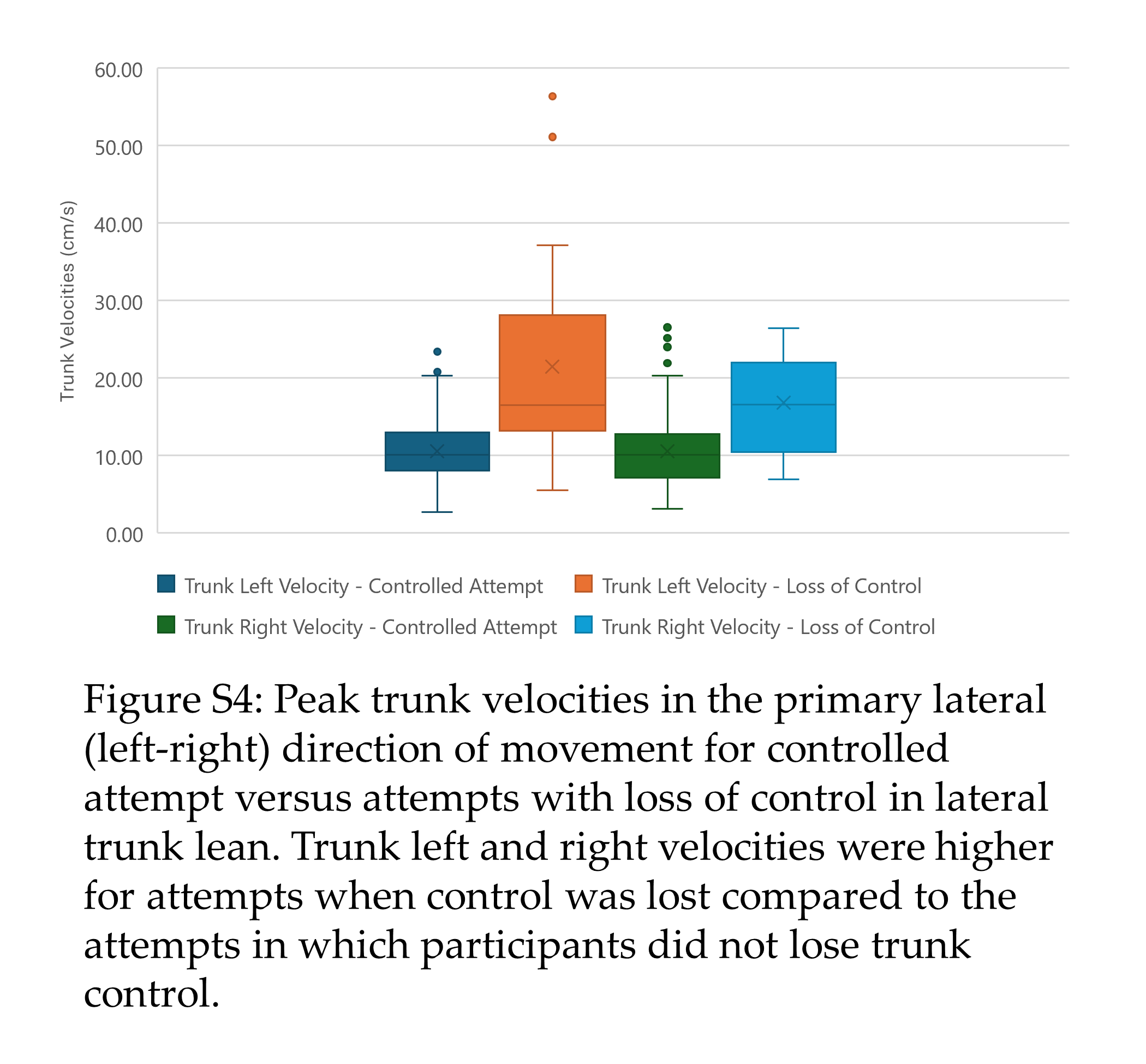

Supplement: Supplementary file 1 [file biomedicines-13-00394-s001.zip › Figure S4.TIF]

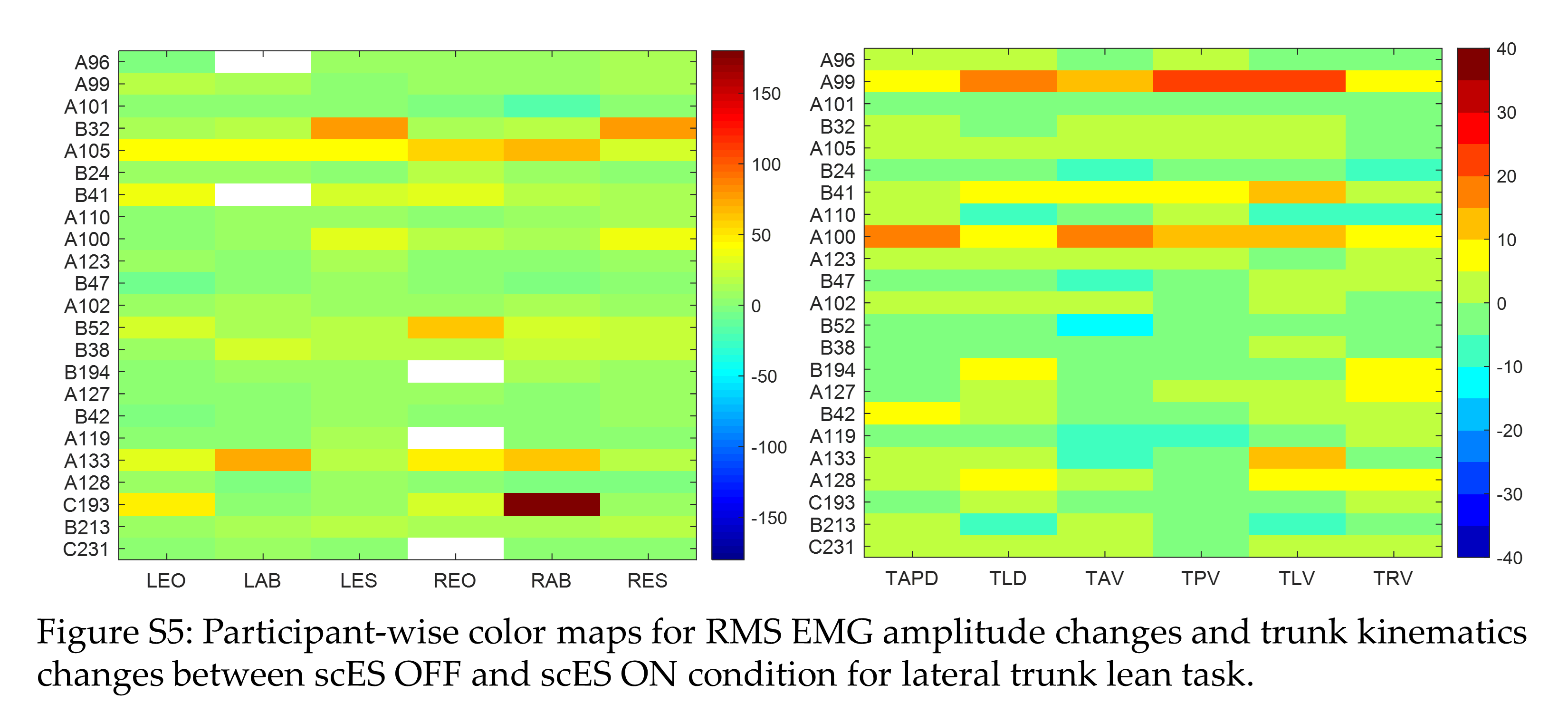

Supplement: Supplementary file 1 [file biomedicines-13-00394-s001.zip › Figure S5.TIF]

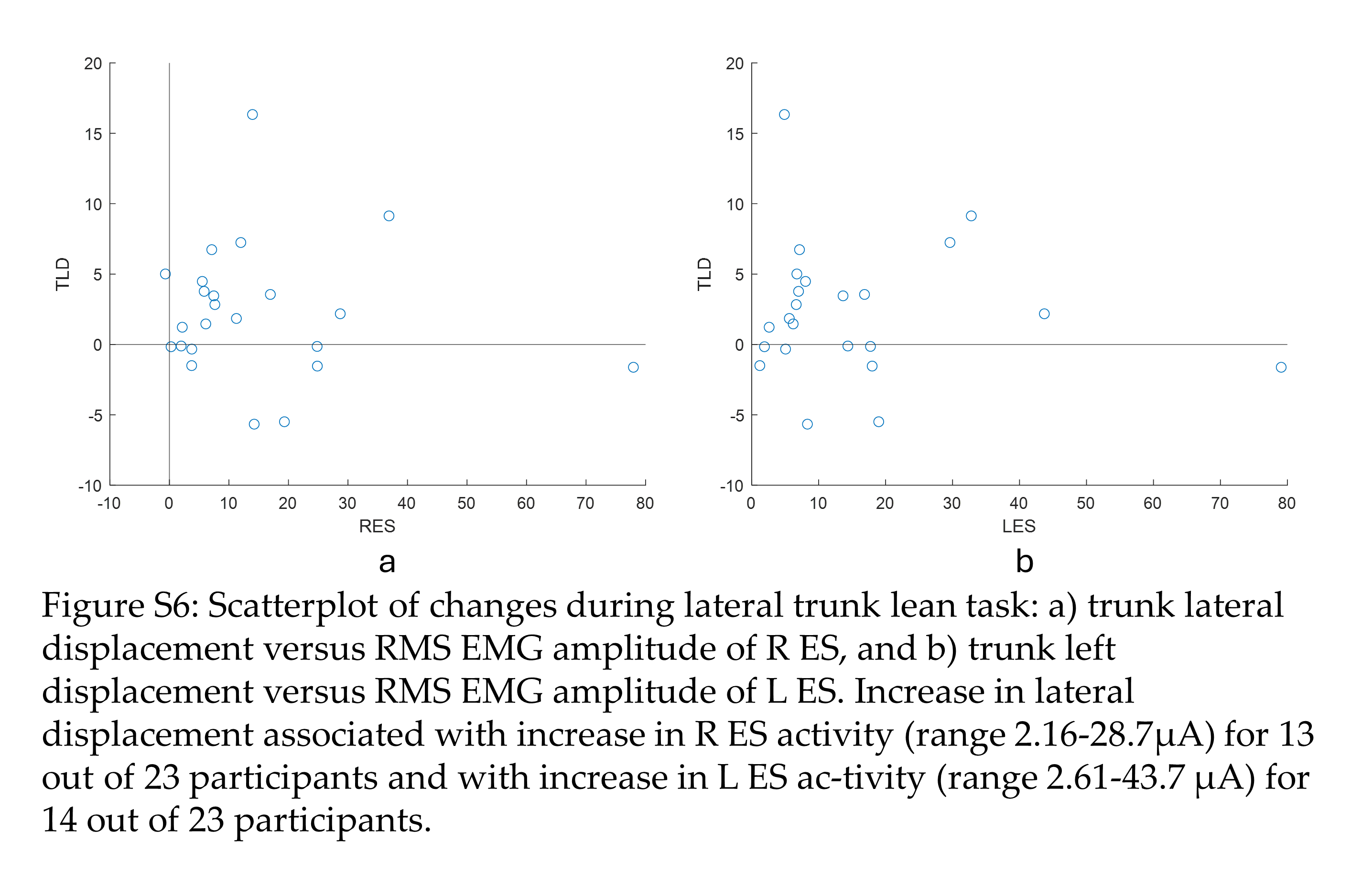

Supplement: Supplementary file 1 [file biomedicines-13-00394-s001.zip › Figure S6.tif]

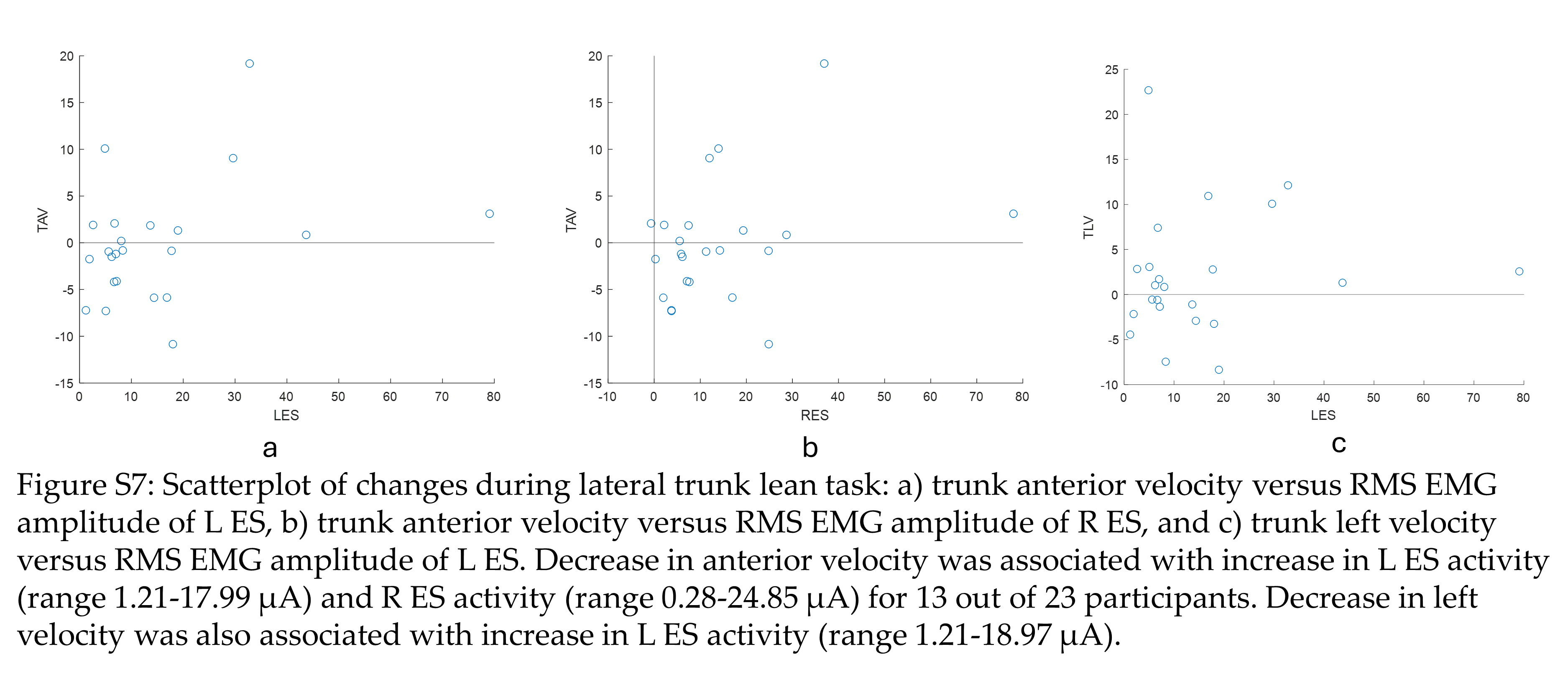

Supplement: Supplementary file 1 [file biomedicines-13-00394-s001.zip › Figure S7.TIF]

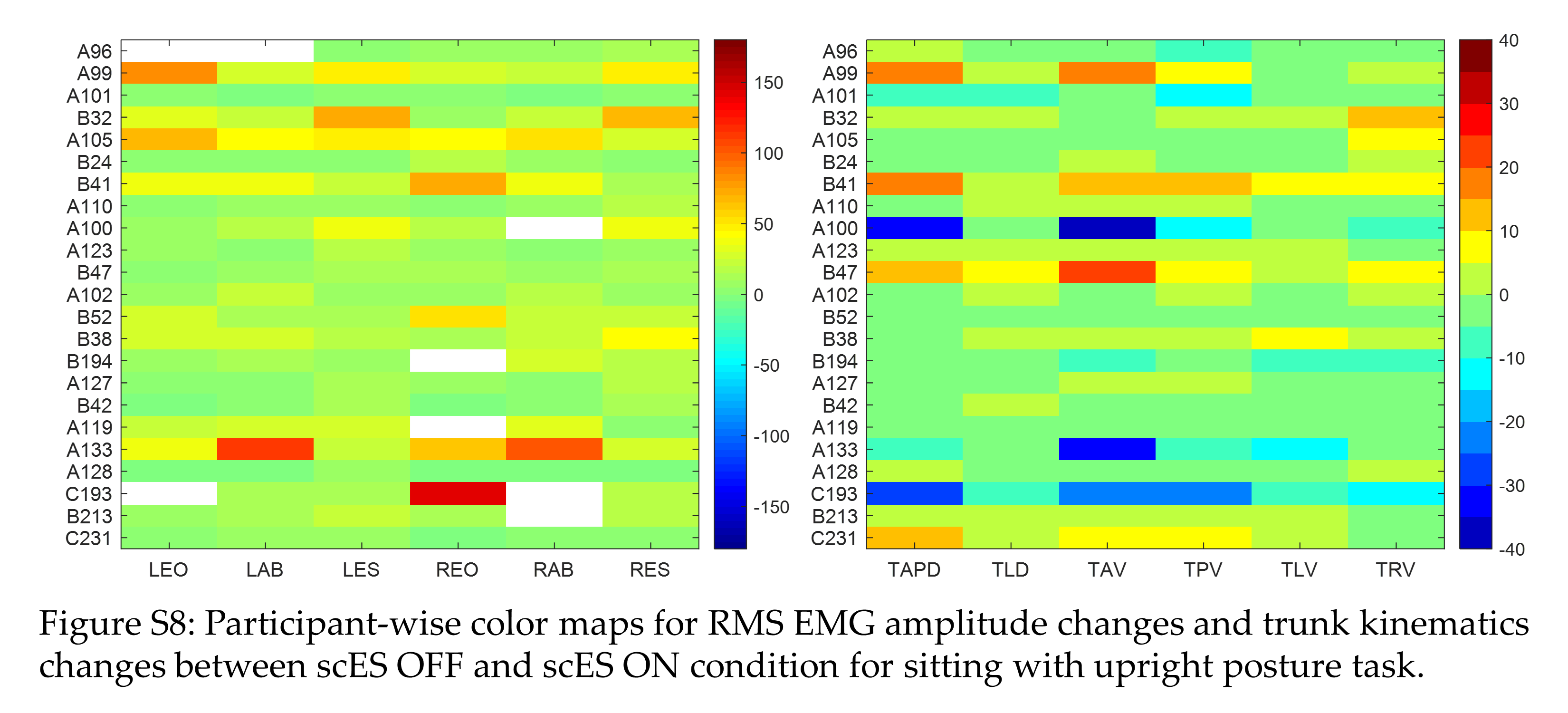

Supplement: Supplementary file 1 [file biomedicines-13-00394-s001.zip › Figure S8.TIF]
